# Compliance Prediction for Structural Topology Optimization on the Basis of Moment Invariants and a Generalized Regression Neural Network

**DOI:** 10.3390/e25101396

**Published:** 2023-09-29

**Authors:** Yunmei Zhao, Zhenyue Chen, Yiqun Dong

**Affiliations:** 1 School of Aerospace Engineering and Applied Mechanics, Tongji University, Shanghai 200092, China; 2230883@tongji.edu.cn; 2 Department of Aeronautics and Astronautics, Fudan University, Shanghai 200433, China

**Keywords:** composite design, topology image recognition, moment invariants, GRNN, compliance

## Abstract

Topology optimization techniques are essential for manufacturing industries, such as designing fiber-reinforced polymer composites (FRPCs) and structures with outstanding strength-to-weight ratios and light weights. In the SIMP approach, artificial intelligence algorithms are commonly utilized to enhance traditional FEM-based compliance minimization procedures. Based on an effective generalized regression neural network (GRNN), a new deep learning algorithm of compliance prediction for structural topology optimization is proposed. The algorithm learns the structural information using a fourth-order moment invariant analysis of the structural topology obtained from FEA at different iterations of classical topology optimization. A cantilever and a simply supported beam problem are used as ground-truth datasets, and the moment invariants are used as independent variables for input features. By comparing it with the well-known convolutional neural network (CNN) and deep neural network (DNN) models, the proposed GRNN model achieves a high prediction accuracy ($R^{2}$ > 0.97) and drastically shortens the training and prediction cost. Furthermore, the GRNN algorithm exhibits excellent generalization ability on the prediction performance of the optimized topology with rotations and varied material volume fractions. This algorithm is promising for the replacement of the FEA calculation in the SIMP method, and can be applied to real-time optimization for advanced FRPC structure design.

## 1. Introduction

Fiber-reinforced polymer composites (FRPCs), which have outstanding material qualities such as high stiffness, strength, and strength-to-weight ratio, are favorable for applications and have gained a lot of attention from both academia and industries [1]. Manufacturing techniques of FRPCs stand for a multidisciplinary subject that requires proper design rules and topology optimizations [2].

Topology optimization is a powerful tool for helping engineers design innovative structures and products, and has already found successful applications in various energy fields [3,4]. In knowledge-driven theories, minimizing the mean compliance and elastic strain of the energy of a structure is the main objective function for the majority of structural topology optimization problems [5]. Entropy generation is often additionally used to assess topology optimization for high-performance structures in aeronautical engineering and automotive systems [6]. Greater efforts are being put into improving the robustness and effectiveness of optimization algorithms, and their applicability has been confirmed [7,8]. With regard to the topology optimization problems, numerous knowledge-driven strategies have been developed to achieve the optimal structure under given load and boundary conditions, manufacturing restraints, and performance objectives. Such models include the solid isotropic material with penalization (SIMP) [9], the level set [10], bi-directional evolutionary structural optimization (BESO) [11], and moving morphable components (MMC) [12,13].

The SIMP method predicts an optimal material distribution within a given design space, and has been considered the most widely used topology optimization scheme to explore optimal structures [14]. The SIMP method stands for one of the typical mesh-based approaches. The reference domain should first be discretized by creating a finite element mesh, followed by a finite element analysis (FEA) performed to obtain the calculation of the compliance for a topology configuration. The traditional methods devoted to topology optimization issues require considerably fine meshes to produce high-resolution designs; as a result, the FEA calculation can be difficult and time-consuming to implement. The cost of FEA computing and evaluating a compliance-based objective function under the volume constraint increases with the complexity (finer) of the finite element discretization.

A high-performance GUP solver is an efficient answer to low computing needs without sacrificing the quality of the structures, especially for large 3D design problems [15]. In addition, many researchers are increasingly interested in applying machine learning techniques to assist optimization procedures of compliance minimization. As numerical modeling naturally complements the ML technique by providing enormous amounts of relevant data, data creation is no longer a challenge in developing effective and robust data-driven models [16,17]. Ref. [18] suggested a K-means algorithm to reduce the dimension of the design variables, thus shortening the computational time. Ref. [19] established an implicit mapping model of shape function between the high resolution and a coarse resolution to reduce the FEA calculation significantly.

There have also been widespread attempts to speed the process of the convergence of the optimization by using powerful neural network (NN) models [20]. Convolutional neural networks (CNNs), a family of artificial neural networks that have gained dominance in a variety of computer vision tasks [21,22], are the most well-established deep learning algorithms in topology optimization tasks. Thus, the topology optimization problem is transformed from a design challenge to an image recognition problem. For the optimized topology configurations, researchers have proposed replacing the FEA calculation module with CNN models [23,24]. Furthermore, CNN models have been combined with generative adversarial network (GAN) algorithms [25] and long short-term memory networks (LSTM) algorithms [17,26] to accelerate the iteration procedures of the SIMP algorithm. The image-based CNN model achieves high accuracy in mapping the topology configuration to its compliance, while the training from scratch is usually time-consuming, and the black-box nature hinders its applicability [22].

In the fields of image processing and computer vision, the geometric features of an image can also be characterized using a set of measurable quantities known as moment invariants [27]. The moment invariants reflect the inherent properties of rotation, translation, and scale invariance. This calculation finds widespread use in tasks such as object recognition, tracking, and image feature extraction. Using invariants as features instead of the image itself, the selection of artificial neural networks can be flexible to replace the CNN model [24,28].

The radial basis function (RBF) has emerged as a variant of the artificial neural network, showing the principal advantages of easy design, good generalization, and faster training. GRNN [29] is one type of RBF, and it showcases the capability of quickly learning and rapidly converging to the optimal regression surface with a large number of datasets [30]. It has been demonstrated that the GRNN algorithm can more efficiently extract valuable information from images while reducing the training cost compared to the CNN models [28].

In this study, towards elucidating the correlation between compliance and optimized topology configurations, we propose a novel deep learning model based on moment invariant analysis of an efficient GRNN neural network. The paper is outlined as follows. Section 2 discusses the design problem and the existing SIMP method, and elaborates on mathematical calculations of moment invariants, along with the architecture of GRNN used in this study. To determine the suitable deep learning model, this paper compares the compliance prediction performance of the GRNN, DNN, and CNN algorithms. The SIMP simulation results are utilized for dataset generation to train the machine learning models. Section 3 evaluates the prediction model to justify the accuracy, efficiency, and reliability, with evaluations and comparison results with existing models presented. Section 4 gives concluding remarks and explains the limitations of the current framework, and future outlooks are also given.

## 2. Method

**Figure 1:**
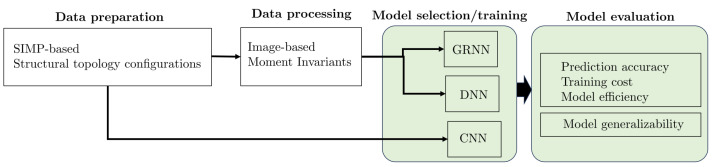
The flowchart of the work.

Figure 1 presents the flowchart of this work, which starts with the utilization of the SIMP method for data creation of structural topology configurations for the design problem. The images are processed to extract their moment invariants, which are then fed into GRNN models as features. In addition to the GRNN model, the CNN and DNN models are trained and optimized for the same design problem. In the last step, the machine learning models are assessed for the performance of accuracy, efficiency, and reliability.

### 2.1. The SIMP Method and Data Preparation

#### 2.1.1. The SIMP Method

As a minimization compliance problem with combinations of variables, the SIMP procedure starts with the discretization of a given domain Ω into a grid of finite elements. A binary value of $\rho_{e}$ is assigned for each element, which is written as:

(1) $$\rho_{e}=\left\{\begin{array}{cc} 0\; & if\;e\in \Omega_{s}\setminus \Omega \\ 1\; & if\;e\in \Omega_{s} \end{array}\right.$$

The value of $\rho_{e}$ describes an element that is either filled with material for regions that require material or emptied of material for regions where material can be removed (representing voids).

The minimization function is subjected to the equilibrium equation and material volume fraction of the optimization material to the original design volume, with consideration of the design variable constraints. The function can be defined as:

(2) $$\underset{\rho }{min}:\;c\left(\rho \right)=U^{T}KU=\sum_{e=1}^{N}\rho_{e}^{p}u_{e}^{T}k_{0}u_{e},\;0<\rho_{min}\leq \rho \leq 1$$

(3) $$subject\;to:\;\sum_{e=1}^{N}v_{e}\left(\rho_{e}\right)/V_{0}=V_{f}$$

(4) KU=F

where *c* is a compliance value, *p* is the penalty factor, and $\rho_{min}$ are lower bounds on densities, which are introduced to refrain from the singularity. *U* and *F* stand for the global displacement and force vectors, respectively. Here, the notation *K* is the global stiffness matrix; $u_{e}$ and $k_{e}$ denote the displacement vector and stiffness matrix of each element. $v_{e}$ is the volume of each element, $V_{f}$ is the target material volume fraction, and *N* is the total number of elements in the design domain ω, which also denotes the resolution of the topology image.

The implementation of SIMP involves multiple iterations. Each iteration produces a topology image, in accordance with four main modules performed: finite element analysis (FEA), analyzing sensitivities (Equations (2)–(4)), filtering sensitivities, and updating design variables, which produce topology images accordingly.

We analyze the time consumption in the SIMP method using the Python performance profiling tool Line profiler [31], as shown in Table 1. The higher computation effort is associated with the modules of FEA calculation and advanced filter techniques, especially when a refined mesh strategy is applied for describing the structural geometry with high resolution.

#### 2.1.2. Data Generation via SIMP

**Figure 2:**
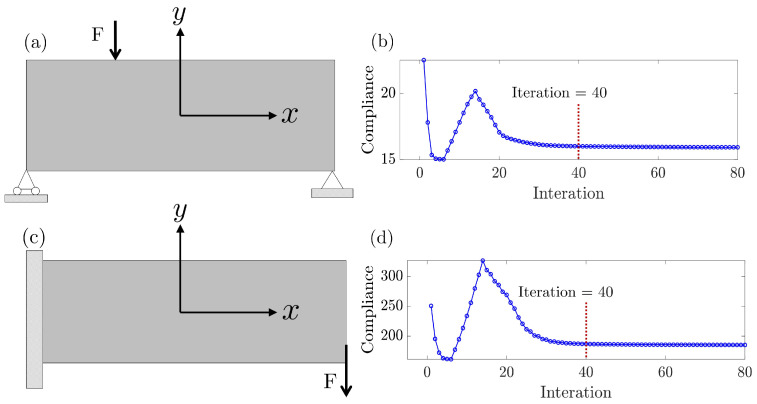
Illustration of topology optimization for two tasks of (**a**) simply supported beam and its compliance iteration plot in (**b**); (**c**) a cantilever beam and its compliance iteration plot in (**d**).

We employ the SIMP algorithm to generate a dataset. As illustrated in Figure 2, two topology optimization tasks are considered, namely the simply supported beam in Figure 2a and the cantilever beam in Figure 2b.

The design domain has a dimension of 120×40, with load *F* as a concentrated force. For each task, the loading position of [$F_{x}$, $F_{y}$] is defined as the distance between the load node and the center of the design domain within the xOy Cartesian coordinates, and material volume fraction $V_{f}$ comprises the primary constraints. For the simply supported beam, the load *F* can be applied to the nodes of the upper and lower surfaces, while the load of a cantilever beam can act on the surface of the free end. The material volume fraction $V_{f}$ follows a normal distribution $f_{0}∼N\left(\mu =0.5,\sigma =0.1\right)$, with a minimum value of 0.176 and a maximum value of 0.802.

As displayed in Figure 2b,d, toward minimizing compliance and obtaining the optimal topology configuration, the implementation of the SIMP algorithm updates the image in response to the FEA calculation in each iteration.

It is noticed that the 40 iterations record the topology images from blurry configurations to the convergence images; thus, we collect the FEA-determined compliances and their topology images at the first 40 iterations to construct the dataset. A similar strategy has been also addressed in [32] for the CNN model training.

In accordance with the SIMP implementations, a dataset is obtained with 20,000 images for the two tasks, in which the dataset’s completeness and diversity are guaranteed.

**Figure A1:**
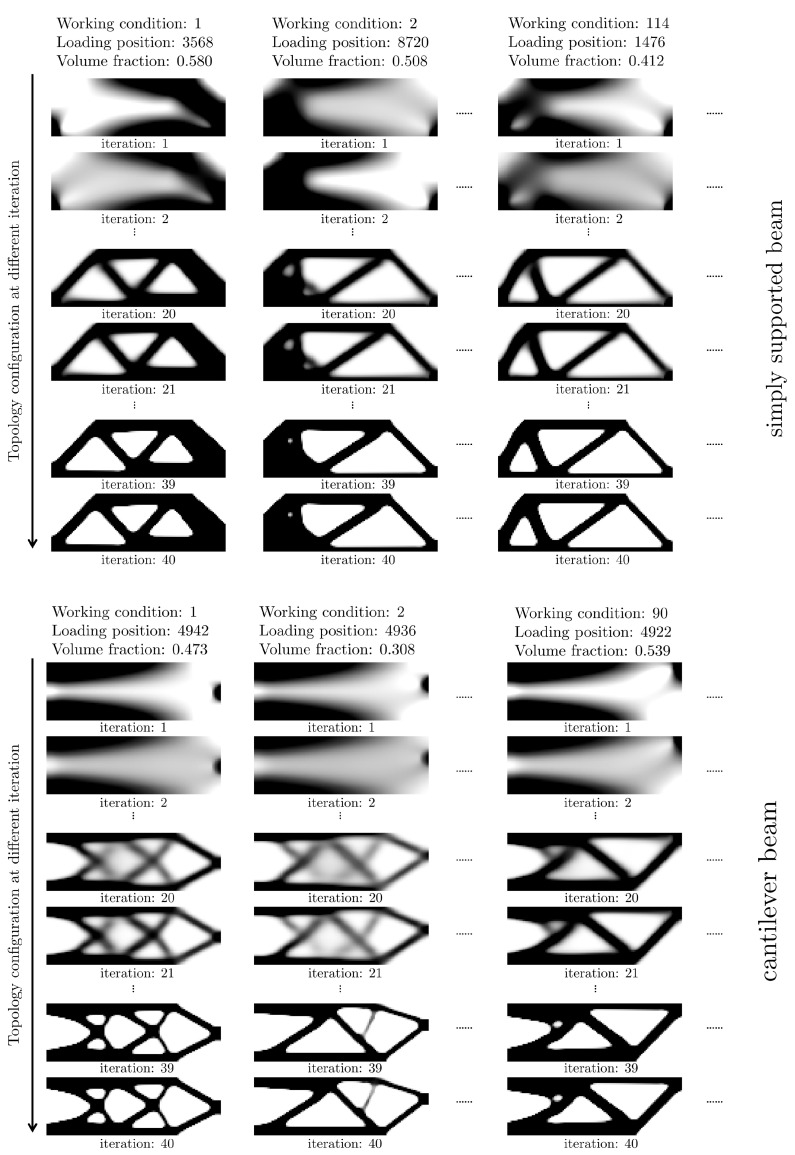
Demonstration of the dataset in accordance with SIMP implementations: showing the constraints and topology configurations at iterations from 0 to 40.

Figure A1 illustrates the image distributions with considered constraints. Of those, 80% and 20% are designated for training and testing, respectively.

### 2.2. Moment Invariants

The fundamental of moment invariants is to extract image information using a set of measurable quantities known as invariants. The geometric moment *m* is the most basic type of moment invariant.

For a two-dimensional image, the p+q order geometric moment is defined as follows:

(5) $$m_{pq}=\int_{-\infty }^{+\infty }\int_{-\infty }^{+\infty }x^{p}y^{q}f\left(x,y\right)dxdy$$

where (*x*, *y*) represents the coordinates of each pixel in the image within a Cartesian coordinate system, and f(x,y) is the density distribution function. Geometric moments can reflect numerous geometric features of the image, capturing the structural characteristics of the topology image. For example, the zero-order geometric moment $m_{00}$ describes the “mass” of the image, which is the area of a binary image. Similar to the “moment of inertia” in material mechanics, $m_{10}/m_{00}$ and $m_{01}/m_{00}$ are the centroid of the image; meanwhile, the second-order moments of $m_{20}$ and $m_{02}$ describe the mass distribution of the image.

Furthermore, the character of shape-preserving transformations like translation, scaling, rotation, and mirroring can be preserved by image invariants. The widely used Hu moments [27] consist of seven independent variables and are derived using only the first three-order geometric moments, which may not be sufficient for extracting enough structural information from topology images.

This study adopts the method derived from [33], which provides a set of invariant moments *M* of arbitrary order that is complete and independent. The calculation of invariant moments *M* can be derived using the complex moment $c_{pq}$, which has the following expression:

(6) $$c_{pq}=\sum_{k=0}^{p}\sum_{j=0}^{q}\left(\begin{array}{c} p\\ k \end{array}\right)\left(\begin{array}{c} q\\ j \end{array}\right){\left(-1\right)}^{q-j}\cdot i^{p+q-k-j}\cdot m_{p+q-k-j}$$

The moment invariants *M* of order *r* are derived from all the complex moments $c_{pq}$ of order p+q, where p+q<r:

(7) $$B=\left\{\begin{array}{c} \Phi \left(p,q\right)\equiv c_{pq}c_{q_{0}p_{0}}^{p-q}\;|\;\left(p\geq q\right)\;\wedge \;\left(2\leq p+q\leq r\right) \end{array}\right\}$$

(8) $$M_{Re}=\left\{\begin{array}{c} m_{Re}=Re\left(\phi \right)\;|\;\phi \in B \end{array}\right\}$$

(9) $$M_{Im}=\left\{\begin{array}{c} m_{Im}=Im\left(\phi \right)\;|\;\left(\phi \in B\right)\wedge \left(p>q\right)\wedge \left(p\neq p_{0}\vee q\neq q_{0}\right) \end{array}\right\}$$

(10) $$M=M_{Re}\cup M_{Im}$$

where *B* is the complex form base set of *r*-order moment invariants *M* in Equation (7). According to Equations (8) and (10), extracting the real and unconjugated imaginary parts of all elements in set *B* constitutes the final representation of the *r*-order invariant moment *M* set.

Among them, $q_{0}$ and $p_{0}$ are indicators that satisfy the conditions for any allowed image. The base set defined by Equation (7) to Equation (10) depends on the selection of $q_{0}$ and $p_{0}$. In practical applications, on the one hand, we hope to keep $q_{0}$ and $p_{0}$ as small as possible, because compared to higher-order moments, lower-order moments are not sensitive to noise. In addition, $c_{q_{0}p_{0}}$ approaching 0 will make the value of the invariant unstable.

In this study for dealing with topology images, based on the algorithm proposed by [33], taking $p_{0}=2$, $q_{0}=1$, and r=4, the set *B* containing seven complex elements is calculated using Equation (7). Then, according to Equation (8), we take the real part of all elements in set *B*, and take the imaginary part of some of them according to Equation (9), to obtain a four-order moment invariants base set *M* containing a total of 11 elements, as described in Equation (11):

(11) $$M=\left\{\begin{array}{c} M_{i}|i=1,2,\cdots ,11 \end{array}\right\}=\left\{\begin{array}{c} M_{1}=Re\left\{\begin{array}{c} c_{11}c_{12}^{0} \end{array}\right\}\\ M_{2}=Re\left\{\begin{array}{c} c_{20}c_{12}^{2} \end{array}\right\}\\ M_{3}=Im\left\{\begin{array}{c} c_{20}c_{12}^{2} \end{array}\right\}\\ M_{4}=Re\left\{\begin{array}{c} c_{21}c_{12}^{1} \end{array}\right\}\\ M_{5}=Re\left\{\begin{array}{c} c_{30}c_{12}^{3} \end{array}\right\}\\ M_{6}=Im\left\{\begin{array}{c} c_{30}c_{12}^{3} \end{array}\right\}\\ M_{7}=Re\left\{\begin{array}{c} c_{22}c_{12}^{0} \end{array}\right\}\\ M_{8}=Re\left\{\begin{array}{c} c_{31}c_{12}^{2} \end{array}\right\}\\ M_{9}=Im\left\{\begin{array}{c} c_{31}c_{12}^{2} \end{array}\right\}\\ M_{10}=Re\left\{\begin{array}{c} c_{40}c_{12}^{4} \end{array}\right\}\\ M_{11}=Im\left\{\begin{array}{c} c_{40}c_{12}^{4} \end{array}\right\} \end{array}\right.$$

### 2.3. Generalized Regression Neural Network (GRNN)

**Figure 3:**
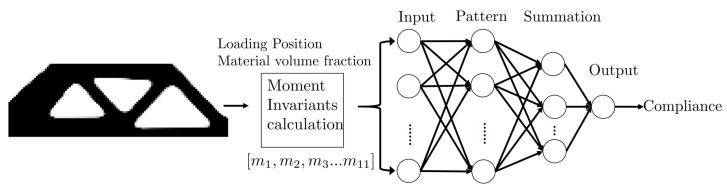
The GRNN framework uses the moment invariants of topology configuration as input features to predict the compliances.

As in Figure 3, the GRNN model has four layers, which are the input layer, pattern layer, summation layer, and output layer. The fundamental principle of GRNN is nonlinear regression analysis [29], and the mathematical formula can be written as:

(12) $$\hat{y}=\frac{\sum_{i=1}^{N}y_{i}exp\left(-\frac{{\left(x-x_{i}\right)}^{T}\left(x-x^{i}\right)}{\sigma^{2}}\right)}{\sum_{i=1}^{N}exp\left(-\frac{{\left(x-x_{i}\right)}^{T}\left(x-x_{i}\right)}{\sigma^{2}}\right)}$$

where $\hat{y}\left(x\right)$ is the weighted average of all sample observations $y_{i}$, and the weight factor of each observation $y_{i}$ is the exponent of the squared Euclid distance between the corresponding sample $x_{i}$ and *x*. σ is the key parameter of the smoothing factor. When σ has a large magnitude, the prediction $\hat{y}\left(x\right)$ approximates the mean of all sample dependent variables, whereas sigma approximating 0 results in a prediction that is very near to the training sample.

### 2.4. Data Processing and Model Training

To obtain a reliable machine-learning-based prediction model, the three neural network models are designed to absorb the images to produce the corresponding compliances. The normalization technique [22] is applied first to process the input features and the predicted compliance.

For compliance prediction tasks, the GRNN model is designed in Figure 3. The topology images are processed to calculate the fourth-order moment invariants as input features using Equation (5) to Equation (10). In addition to the 11-moment invariants, the position of the loading position of $\left(F_{x},F_{y}\right)$ and material volume fraction $V_{f}$ are also chosen as the input feature of GRNN.

**Figure A2:**
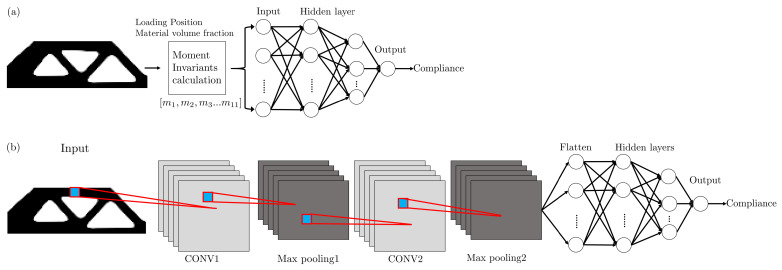
Illustration of model architecture for neural network models of (**a**) DNN and (**b**) CNN.

The input–output pairs for the DNN and CNN model, with the model structure, are detailed in Figure A2. It can be learned that the layer structure of the DNN model is different from the GRNN model, but it is intended to use the same input information as the GRNN model. The CNN model is designed to automatically and adaptively learn topology image information and to predict the compliance, with which the layer structure, parameters, and optimization techniques are used from [23].

The platform for creating datasets is configured with topy 0.4.0 in Python2.7. The network training platform is configured with pyGRNN 0.1.2, scikit-learn 1.0.2, CUDA 11.6, and torch 1.13.1+cu116 with Nvidia driver version 512.36 (GPU: Nvidia RTX3060).

## 3. Results and Discussion

### 3.1. Moment Invariant Calculations

**Figure 4:**
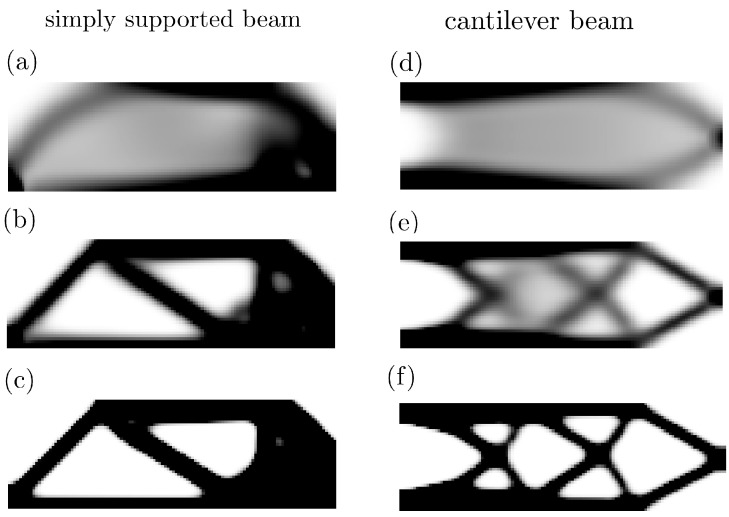
Calculation of moment invariants with six classic structural topology configurations, which include the simply supported beam in (**a**–**c**) and the cantilever beam in (**d**–**f**) at iterations of 10, 20, and 40.

The moment invariant is a feature extraction technique used to extract the global features for shape recognition and identification analysis. Figure 4 gives several classic topology configurations obtained from the dataset. The calculated 11-moment invariants for each case are summarized in Table 2. The 11 independent variables can cover the structural information of translation, scaling, and rotational under a set of loading conditions and material volume constraints.

For developing a deep learning-based image recognition algorithm, the moment invariants are much more efficient compared to the traditional image-processing techniques in CNN models.

### 3.2. Model Performance Evaluation

#### 3.2.1. Model Accuracy

For regression problems, the loss functions mean squared error (MSE) and coefficient of determination $R^{2}$ are used to evaluate the model’s performance.

The MSE and $R^{2}$ can be computed as:

(13) $$MSE=\frac{1}{n}\sum_{n}^{i=1}{\left(\hat{y_{i}}-y_{i}\right)}^{2}$$

(14) $$R^{2}=1-\frac{\sum {\left({\hat{y}}_{i}-y_{i}\right)}^{2}}{\sum {\left(\bar{y}-y_{i}\right)}^{2}}$$

where ${\hat{y}}_{i}$ is the predicted values of compliances, $y_{i}$ is the ground-truth, and $\bar{y}$ is the average value of all compliances.

**Figure 5:**
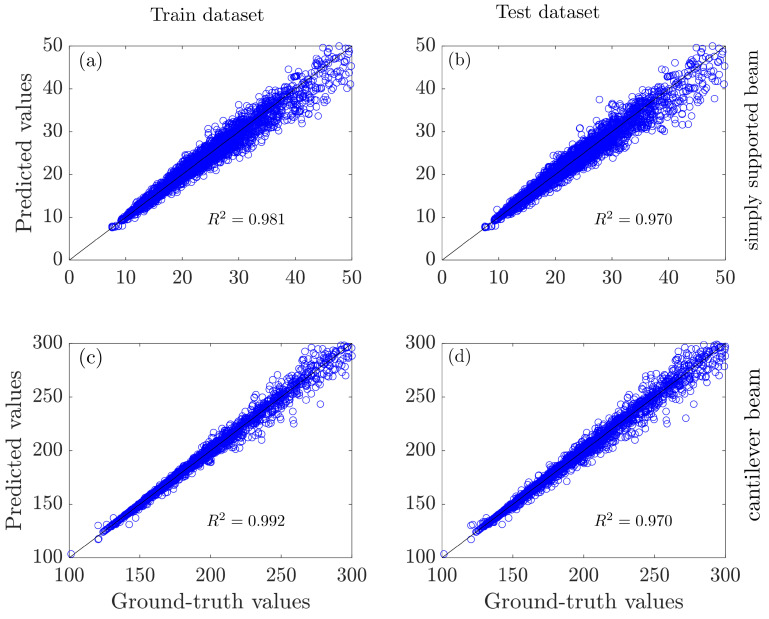
Evaluation of the GRNN performance (**a**) on simply supported beams with the training set and (**c**) the testing set; (**b**) on cantilever beams on the training set and (**d**) testing set.

Since the model is trained on the dataset of the simply supported beam and the cantilever beam, Figure 5 first evaluates the performance of the GRNN model on the two tasks within the dataset. For the simply supported beam, the GRNN model achieves 0.981 and 0.970 on the training and testing datasets, respectively. The magnitudes are 0.992 and 0.970 on the cantilever beam.

**Figure 6:**
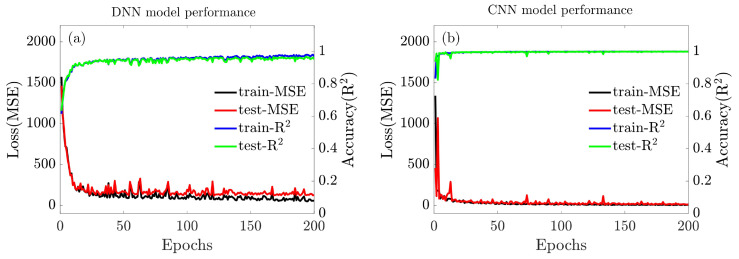
Plots of MSE and $R^{2}$ with epochs of the (**a**) DNN model and (**b**) CNN model.

Figure 6 offers a better insight into the learning performance of the DNN and CNN models by displaying graphs of loss and accuracy over each epoch for training and testing operations. It can be found that compared to the DNN model, the CNN model presents as a very powerful and efficient model that performs automatic feature extraction to achieve high accuracy in predicting topology compliance.

#### 3.2.2. Model Efficiency

The finalized model parameters for GRNN are summarized in Table 3. Additionally, the parameter settings for DNN and CNN models are summarized in Table A3 and Table A4 in Appendix B, respectively.

Table 4 summarizes the prediction accuracy on training and testing sets and the computational efficiency of the three neural networks. In terms of prediction accuracy, it can be shown that all three models perform quite well with the $R^{2}$ higher than 0.96, with the GRNN model reaching a high precision of 0.998 and 0.994 on the training and testing datasets, respectively. The magnitudes are marginally lower than the CNN model, which achieves an $R^{2}$ of 0.999.

As for computational cost, the GRNN significantly overwhelms the other two neural network models. In particular, the training completion time for the GRNN algorithm is 1191.35 s, which is 30 times faster than the CNN model’s (38,662.40 s) time. Moreover, the well-trained GRNN model predicts topology compliances in 0.0019 s, which is a significant improvement over the CNN model’s 2.71 s. In comparison to the GRNN model, the DNN model achieves equivalent accuracy while having a training cost that is roughly 7 times higher and a prediction time that is 3 times slower. Overall, the GRNN model excels at obtaining high precision in predicting the compliances with the lowest computational cost.

It is evident that the well-known CNN model is a very effective tool for automatically extracting features for dealing with image recognition and segmentation problems, but it always comes with a significant training burden. As seen in Table A4, training a CNN model necessitates thousands of parameters. On the one hand, this raises the expense of training, and on the other, it turns the model into a black box with limited interpretability [22]. In contrast, the DNN model in Table A3 has a simpler layer structure and fewer parameters, which reduces training costs but makes certain accuracy tradeoffs.

Regarding the GRNN model in Table 3, it holds the simplest layer structure, with only one hyperparameter (σ), which is the major advantage over other types of NN models. Note that we explore a range of σ values before settling on the magnitude of 0.023, as in Table 3. Meanwhile, it maintains the highest accuracy attributed to the moment invariant analysis. With a large number of datasets, the GRNN model also has the intrinsic capacity to learn rapidly and quickly converge to the best parameter; therefore, it offers the lowest training cost and produces predictions quickly.

### 3.3. Model Generalizability

Using the SIMP algorithm, varied structural topology configurations are generated as unknown samples to examine the generalizability of the trained GRNN model.

#### 3.3.1. Generalizability on Rotated Topology Configurations

As presented in Table 5 and Table 6, for the samples of simply supported beams and cantilever beams, the compliances predicted from the three neural network models are compared with the FEA-determined ground-truth values.

For each task, we select four configurations (specified as images [$0^{\circ }$]) that involve diverse constraints information of loading positions, material volume fractions, and iteration steps. Accordingly, for the simply supported beams, the FEA-determined structural compliances are 12.086, 23.871, 16.116, and 20.538, with the constraint details being found in Table A1 in the Appendix A. For the cantilever beams, the FEA-determined compliances are 213.518, 532.576, 228.132, and 168.211, with the constraints listed in Table A2 in the Appendix A.

Firstly, the three models demonstrate comparable generalization capability on the topology configurations (images [$0^{\circ }$]), while the GRNN model exhibits the best performance with a maximum relative error of less than 3.5%. Furthermore, when rotating those images clockwise 90, 180, and 270 degrees, it is worth noting that the time-consuming CNN model fails to learn the geometry transformations with a prediction error greater than 91%. In contrast, for the GRNN and DNN models, the rotation-invariance property based on the moment invariant analysis enables the network’s capability to predict the compliances regardless of the orientations. Thus, the predicted outcomes are the same compared to the configurations before rotation (images [$0^{\circ }$]).

**Figure 7:**
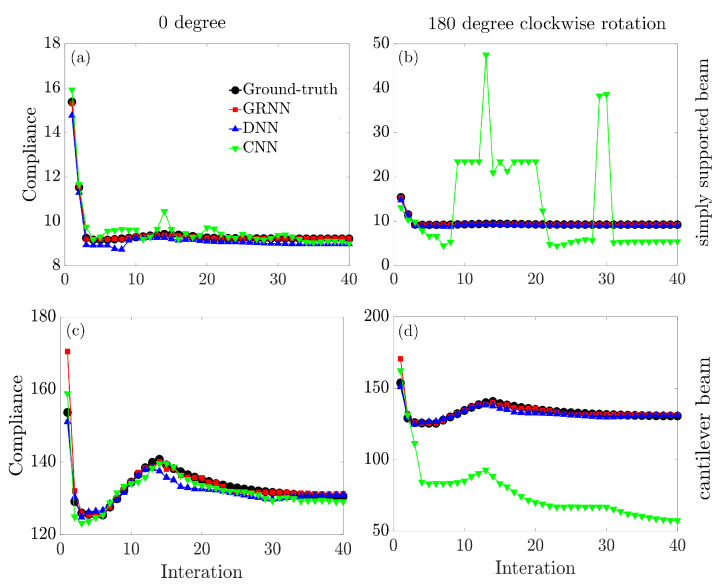
Model generalization on a simply supported beam with the topology configuration (**a**) 0 degree and (**b**) 180 degree clockwise rotation; and on a cantilever beam with the topology configuration (**c**) 0 degree and (**d**) 180 degree clockwise rotation.

Figure 7 further explores the model predictions along with iterations from 0 to 40. It can be seen that the developed GRNN model accurately captures the nature of minimization of compliance along the iterations. The comparison demonstrates another advantage of using moment invariants to extract structural information and then employing GRNN for predicting compliances. When the topological configurations are rotated, it appears that the CNN model entirely loses its ability to predict. However, GRNN may still sustain a comparatively high level of prediction accuracy owing to the rotational invariance of the moments.

#### 3.3.2. Generalizability on Topology Configurations with Different Material Volume Fractions

It is known that the topology optimization follows compliance minimization criteria when subject to mechanical constraints of a certain load and material volume fraction. There are a handful of studies that explain that an increased material volume fraction produces a decreased compliance magnitude [34].

**Figure 8:**
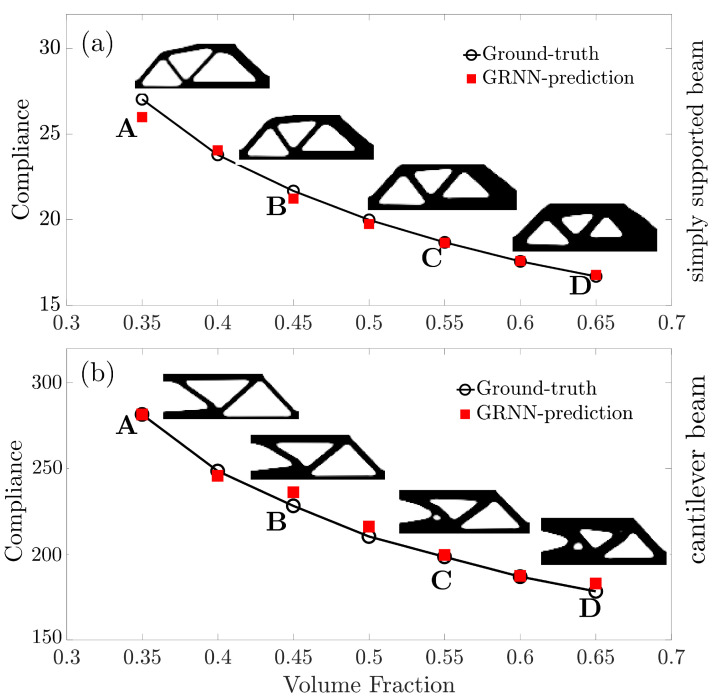
Investigation of the GRNN predicted compliance with different volume fractions for (**a**) a simply supported beam and (**b**) a cantilever beam.

Figure 8 plots the predicted compliance in response to changed material volume fraction $V_{f}$ for the two topology optimization tasks. It can be found that the underlying mechanism of compliance variation and the input feature of material volume fraction is accurately learned by the GRNN model. The GRNN model exhibits outstanding generalization ability for predicting compliance in response to changed material volume fraction.

## 4. Conclusions and Outlook

In this work, a novel GRNN algorithm is developed to predict the topology compliance, together with the moment invariants as independent variables, which are obtained from the FEA-determined structural topology. The algorithm is trained using data produced by the SIMP approach, which takes into account two classic tasks—a cantilever beam and a simply supported beam—at various iteration stages. With a comparison to CNN and DNN models, the model is thoroughly evaluated using the metrics of accuracy, efficiency, and generalizability. Through the study, the following conclusions can be drawn.

(1) The prediction accuracy of the GRNN model in the training and testing sets is supported by $R^{2}>0.97$ and drastically shortens the training and prediction cost compared to the CNN and DNN models.

(2) Compared with the DNN and CNN algorithms, the GRNN model shows the best generalization ability on compliance prediction for structural topology with rotations and under different material volume fractions.

(3) The moment invariants have translation, scaling, and rotational invariance, and the evaluation demonstrates that using the moment invariants as features is important in determining a reliable deep learning model for compliance prediction in topology optimization problems.

In this study, the GRNN is considered a preferred algorithm with high accuracy, efficiency, and generalization ability in the application of exploring the underlying relationship between the structural topology and its compliance. However, we still acknowledge several limitations. The current work denotes an end-to-end paradigm to predict the structural compliances in the SIMP method, which represents a simplified classic isotropic scenario without considering the manufacturing constraints for fiber-reinforced composites. The generalizable deep learning method represents a potential strategy in aspects of replacing the FEA calculation for high-resolution problems within the traditional topology optimization framework. Future efforts can be improved by involving constraints of the placement of path planning of fibers, which is essential in customizing the mechanical anisotropy in manufacturing, with the traditional numerical implementation being more costly.

The current work is promising in that it can be extended to the revised design issues and generate real-time optimization for advanced FRPC structure design [35,36]. It can also be utilized together with fiber printing techniques for applications in additive manufacturing [37].

## Figures and Tables

**Table 1:** Computing proportion for each module of the SIMP method.

Module in SIMP	Computational Cost
FEA	30.3%
Sensitivities	0.1%
Filter	67.4%
Variables update	2.2%

**Table 2:** Calculations of moment invariants of different topology configurations as in Figure 4.

Moment Invariants	Case a	Case b	Case c	Case d	Case e	Case f
m1	0.011625	0.011821	0.011668	0.010311	0.010683	0.010505
m2	0.000767	0.000752	0.000832	0.000416	0.000458	0.000481
m3	0.000499	0.000506	0.000535	−0.000240	−0.000278	−0.000276
m4	0.000237	0.000228	0.000266	0.000097	0.000110	0.000119
m5	0.000229	0.000222	0.000256	0.000101	0.000115	0.000124
m6	−0.000106	−0.000126	−0.000145	−0.000057	−0.000065	−0.000066
m7	0.000166	0.000169	0.000169	0.000125	0.000130	0.000129
m8	0.000194	0.000190	0.000211	0.000099	0.000109	0.000114
m9	0.000120	0.000123	0.000130	−0.000057	−0.000065	−0.000065
m10	0.000179	0.000173	0.000198	0.000086	0.000096	0.000102
m11	0.000152	0.000152	0.000166	−0.000071	−0.000081	−0.000083

**Table 3:** Parameter settings for the GRNN model, with the spread σ being the only hyperparameter.

P (Input Vectors)	T (Target Class Vectors)	Spread σ
[15,242×14]	[15,242×1]	0.023

**Table 4:** Model performance evaluation of accuracy ($R^{2}$) and computational cost of the three neural networks.

Model	Prediction Accuracy		Computational Efficiency	
	**Training Set**	**Testing Set**	**Training**	**Prediction**
GRNN	0.998	0.994	1191.35 s	0.0019 s
DNN	0.991	0.964	7128.01 s	0.0061 s
CNN	0.999	0.999	38662.40 s	2.71 s

**Table 5:** Model generalization on unknown samples of simply supported beams.

Images [0°]	FEA Calculation	Compliance Predictions					
		GRNN	DNN	DNN			
				[0°]	[90°]	[180°]	[270°]
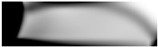	12.086	12.128	11.842	11.516	26.555	22.240	55.067
		0.352%	2.017%	4.716%	119.719%	84.013%	355.633%
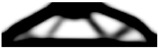	23.871	24.684	24.758	23.297	30.567	11.785	33.911
		3.408%	3.716%	2.405%	28.051%	50.630%	42.062%
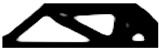	16.116	16.127	15.923	15.627	20.688	23.765	50.847
		0.064%	1.020%	3.035%	28.364%	47.464%	215.516%
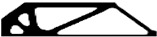	20.538	20.607	21.134	19.826	38.330	14.006	30.903
		0.334%	2.753%	3.468%	86.626%	31.806%	50.465%

**Table 6:** Model generalization on unknown samples of the cantilever beams.

Images [0°]	FEA Calculation	Compliance Predictions					
		GRNN	DNN	DNN			
				[0°]	[90°]	[180°]	[270°]
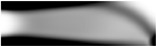	213.518	208.998	206.768	213.867	39.191	339.148	39.817
		2.117%	3.161%	0.163%	81.645%	58.838%	81.352%
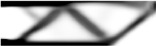	532.576	546.824	515.810	537.995	30.539	90.565	35.502
		2.675%	3.148%	1.017%	94.266%	82.995%	93.334%
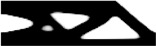	228.132	228.900	232.161	225.368	12.440	110.099	19.820
		0.337%	1.766%	1.211%	94.547%	51.739%	91.312%
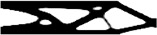	168.211	168.694	167.335	167.391	10.939	90.536	13.731
		0.287%	0.521%	0.488%	93.497%	46.177%	91.837%

## Data Availability

The data that support the findings of this study are available upon reasonable request from the corresponding authors.

## Appendix A. Topology Configurations Used for Model Generalization Evaluation

**Table A1:** The loading position, material volume fraction, and iteration of the topology configurations in accordance with Table 5.

Images $[0^{\circ }$]	Load Position	Material Volume Fraction	Iteration
	329	0.4510	10
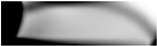
	2297	0.5611	20
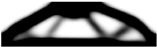
	3608	0.6206	30
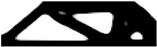
	1148	0.3738	40
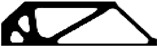

**Table A2:** The loading position, material volume fraction, and iteration of the topology configurations in accordance with Table 6.

Images $[0^{\circ }$]	Load Position	Material Volume Fraction	Iteration
	4958	0.5143	10
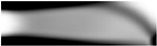
	4924	0.3328	20
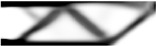
	4960	0.6766	30
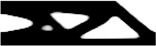
	4948	0.5483	40
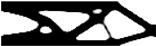

## Appendix B. Deep Neural Networks of DNN and CNN

The DNN model absorbs 14 features as inputs and it is finalized with three hidden layers with the batch size setting as 71, the learning rate setting as 0.0001, and the epoch setting as 400. The CNN model treats it as a topology image recognition problem and takes the image with a resolution of 210×543. As described in [23], the model has a batch size of 32, a learning rate of 0.001, and epochs of 300, with the structure finalized with two convolutional layers and two max-pooling layers, and three hidden layers coming after the flatten layer. For DNN and CNN models, the number of parameters for each input and output layer within its structure is summarized in Table A3 and Table A4, respectively.

**Table A3:** Parameter settings of the DNN neural network model.

Layer (Type)	Input Shape	Output Shape
Input	14	248
Hidden1	248	253
Hidden2-1	253	253
Hidden2-2	253	253
Hidden2-3	253	253
Output	253	1

**Table A4:** Parameter settings of the CNN neural network model.

Layer (Type)	Input Shape	Output Shape
CONV1	121,632	3,600,896
Max pooling1	3,600,896	898,560
CONV2	898,560	1,725,888
Max pooling2	1,725,888	425,600
Flatten	425,600	425,600
Hidden1	425,600	400
Hidden2	400	200
Hidden3	200	1
